# An Ultra-Narrowband Graphene-Perfect Absorber Based on Bound States in the Continuum of All-Dielectric Metasurfaces

**DOI:** 10.3390/nano15141124

**Published:** 2025-07-19

**Authors:** Qi Zhang, Xiao Zhang, Zhihong Zhu, Chucai Guo

**Affiliations:** College of Advanced Interdisciplinary Studies & Hunan Provincial Key Laboratory of Novel-Optoelectronic Information Materials and Devices, National University of Defense Technology, Changsha 410073, China

**Keywords:** graphene, ultra-narrowband, perfect absorption

## Abstract

Enhancing light absorption in two-dimensional (2D) materials, particularly few-layer structures, is critical for advancing optoelectronic devices such as light sources, photodetectors, and sensors. However, conventional absorption enhancement strategies often suffer from unstable resonant wavelengths and low-quality factors (Q-factors) due to the inherent weak light–matter interactions in 2D materials. To address these limitations, we propose an all-dielectric metasurface graphene-perfect absorber based on toroidal dipole bound state in the continuum (TD-BIC) with an ultra-narrow bandwidth and stable resonant wavelength. The proposed structure achieves tunable absorption linewidths spanning three orders of magnitude (6 nm to 0.0076 nm) through critical coupling modulation. Furthermore, the operational wavelength can be flexibly extended to any near-infrared region by adjusting the grating width. This work establishes a novel paradigm for enhancing the absorption of 2D materials in photonic device applications.

## 1. Introduction

Since the discovery of graphene through mechanical exfoliation in 2004 [1], it possesses numerous unique and excellent physical properties, such as high carrier mobility, high thermal conductivity, a broad spectral absorption range, tunable Fermi level, and outstanding mechanical properties [2,3]. These characteristics endow graphene with broad application prospects in numerous fields, such as micro-light sources [4,5,6,7], perfect absorbers [8,9], sensors [10], photodetectors [11,12], and modulators [13,14]. However, their atomic thickness typically results in low light absorption (for instance, monolayer graphene exhibits an absorption rate of only 2.3% in the near-infrared band) [15]. This weakens the interaction between light and 2D materials, thereby limiting their applicability in various scenarios. Various approaches have been explored to enhance the absorption of 2D materials [16], including surface plasmon resonance [12,17,18,19], Fabry–Pérot cavities [11], and metamaterials [20,21]. Among them, metal surface plasmon excitations can achieve perfect absorption of graphene but inevitably introduce ohmic losses. Fabry–Pérot cavities require multiple dielectric layers for high absorption, often involving complex preparation processes. In contrast, all-dielectric meta-surfaces consisting of artificial subwavelength structures have gained attention for significantly enhancing light–matter interactions at the nanoscale [8,22,23]. Particularly, bound states in the continuum (BICs)—characterized by infinite radiative lifetimes and ultrahigh quality factors—have enabled breakthroughs in laser design [24,25], nonlinear optics [26,27,28], and importantly, 2D material absorption enhancement through optimized mode coupling [21,29,30,31,32]. This approach addresses previous limitations while maintaining material integrity.

BICs are eigenstates with forbidden radiation that are completely decoupled from their external environment, despite existing in the continuum domain [33,34]. A BIC must be converted into a quasi-BIC (q-BIC) with a finite yet high-quality factor (Q factor) by breaking the symmetry of the excited unit to realize practical applications [27,35,36]. Here, the Q factors are inversely proportional to the square of the structural asymmetry parameter [37,38,39]. Previous studies on BIC-coupled 2D material systems predominantly focused on geometric parameter optimization under critical coupling conditions to enhance absorption [29,30]. Nevertheless, such approaches require stringent control over structural geometry, where minor variations in asymmetry inevitably modify the effective refractive index, resulting in substantial shifts in resonance wavelength that compromise wavelength stability [40,41,42,43]. Although recent advances demonstrate that the toroidal dipole BIC (TD-BIC) in composite lattice structures are beneficial for wavelength-stabilized graphene absorbers, the inherent absorption loss of graphene fundamentally limits this approach [21]. Thus, these graphene-based perfect absorbers based on BIC require strategies that suppress structural radiation loss. This requirement inherently causes degradation of their Q factors.

Currently, practical implementations of BIC-enhanced absorption in two-dimensional materials face two primary challenges: (i) the dependence of resonance wavelength on perturbation parameters and the selective excitation of specific resonant wavelengths and (ii) the inherent absorption loss compromising Q-factor performance when maximizing absorption through temporal coupled-mode theory (TCMT). Recent research has explored the robustness of the Q-factor and achieved the stable resonance wavelength by employing Brillouin zone folding BIC (BZF-BIC) [28,34,44], strategic symmetry breaking [39,45], and adding a perturbation layer matching the refractive index of the substrate [46]. However, the fundamental conflict between achieving simultaneous high absorption efficiency and high Q-factor performance persists, particularly for practical device applications. This unresolved challenge underscores the critical importance of developing ultra-narrowband perfect absorbers based on graphene and other 2D materials.

In this work, we present a BIC-based composite grating structure to realize an ultra-narrowband (or high Q-factor) graphene-perfect absorber. By precisely tailoring the silicon grating gap dimensions and silica overlayer thickness, ultra-narrowband perfect absorption in graphene is achieved through critical coupling conditions. Keeping the grating width constant allows the graphene-perfect absorber to maintain a stable peak wavelength as the grating gap varies. Additionally, by varying the grating width, high Q-factor graphene-perfect absorbers can be achieved at arbitrary wavelengths. This method can be extended to applications such as detecting the characteristic spectra of various 2D materials.

## 2. Materials and Methods

**Figure 1:**
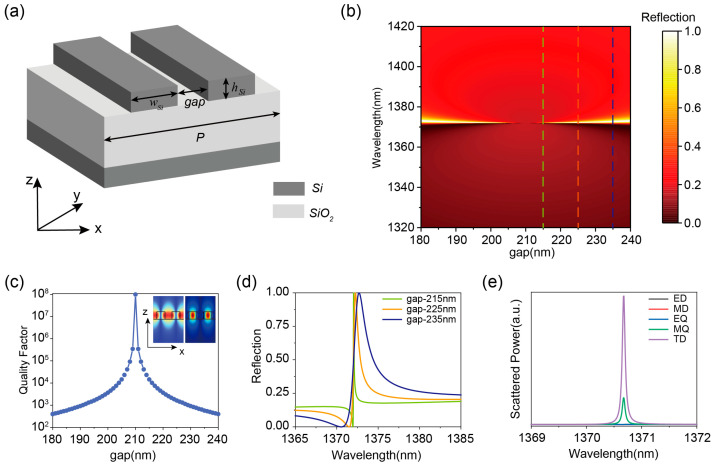
(**a**) Schematic structure of the silicon double grating composite structure. (**b**) Reflection spectra of the composite structure at different grating gaps. (**c**) Variation of the Q-factor of the composite structure at different grating spacings. The inset shows the intrinsic electric and magnetic field contours of the composite structure at resonant wavelengths. (**d**) Reflection spectra of the composite structure extracted from (**b**) at gaps of 215 nm, 225 nm, and 235 nm. (**e**) Multipole decomposition at resonant wavelengths.

To achieve a q-BIC with a stable resonance wavelength, an all-dielectric meta-surface through a composite grating structure is proposed, as illustrated in Figure 1a. The structure employs a silicon-on-insulator (SOI) platform for simplified fabrication, where translation-symmetry-protected BIC modes emerge through controlled lateral displacement of constituent gratings. The period (*P*) of the composite grating during the study is 900 nm, the width (*w_Si_*) and the height (*h_Si_*) of the grating are 240 nm and 220 nm, respectively, and the only variable in the structure is the gap of the composite grating. The structure supports multiple leakage modes. Among the many leak modes, this TD BIC built on the TE_31_ mode allows for the transition from BIC to quasi-BIC without breaking structural symmetry, resulting in a stable resonance wavelength while tailoring the quality factor by varying the gap distance. Therefore, here we focus on the TE_31_ mode, whose intrinsic electric and magnetic field profiles are illustrated in the Figure 1c inset.

## 3. Results

The Q factors in Figure 1b are calculated by the finite element method of commercial 3D finite element software (COMSOL Multiphysics 6.0). The refractive indices of Si and SiO_2_ are set as 3.48 and 1.46, respectively. The results are shown in Figure 1c, and it is found that the leakage mode converts to BIC when the grating gap is the critical value *gap*_0_ (210 nm), and BIC converts to q-BIC when the grating deviates from this critical value. To verify the BIC existence, the variation of the reflectance spectra of the composite grating structure as a function of the grating pitch spacing is calculated, as shown in Figure 1b. The vanishing of optical resonance linewidth in reflectance spectra at the critical gap (*gap*_0_) confirms BIC formation. At this point, the center distance between the two nano gratings is 450 nm, which is exactly half of the period of the composite grating.

This phenomenon can be interpreted as a Brillouin zone folding BIC (BZF-BIC), where the band-edge X-points in the first Brillouin zone of a single grating (period *P*/2) are folded into the Γ-point of the composite grating’s first Brillouin zone [33,34,44,47]. Notably, the resonance wavelength of such symmetry-broken BICs exhibits insensitivity to asymmetric parameter variations [33,34], making this mechanism particularly effective in alleviating fabrication-induced imperfections in practical implementations.

Furthermore, when the grating gap deviates from the critical value, the q-BIC exhibits a Fano resonance profile characterized by spectral broadening and Q-factor degradation [48,49,50,51], as systematically demonstrated in Figure 1c,d. This parametric evolution reveals an inverse proportionality between structural detuning (Δ*g* = |*gap* − *gap*_0_|) and resonance quality. As the gap increases from 215 nm to 235 nm (corresponding to ∆*g* increasing from 5 nm to 15 nm), the resonance linewidth broadens from 0.093 nm to 2.36 nm, while the Q-factor dramatically decreases from 1.45 × 10^4^ to 5.72 × 10^2^, demonstrating a symmetry-breaking-induced radiative loss mechanism. Multipolar decomposition analysis in Figure 1e confirms the toroidal dipole (TD) dominance in the q-BIC’s optical response, which is consistent with established theoretical frameworks [21,35].

In previous studies on enhancing light absorption in two-dimensional (2D) materials through meta-surface integration, researchers typically position the 2D material at the maximum electric field intensity to achieve absorption enhancement under critical coupling conditions (*Q_rad_* = *Q_abs_*) [12,21]. However, since the intrinsic absorption loss (*Q_abs_*) of 2D materials under this configuration solely depends on their complex refractive index components: *Q_abs_* = *n_real_*/2*n_imag_* (where *n_real_* and *n_imag_* are the real and imaginary parts of the absorbing material, respectively), implementing perfect absorption in high-Q meta-surface architectures remains fundamentally challenging.

**Figure 2:**
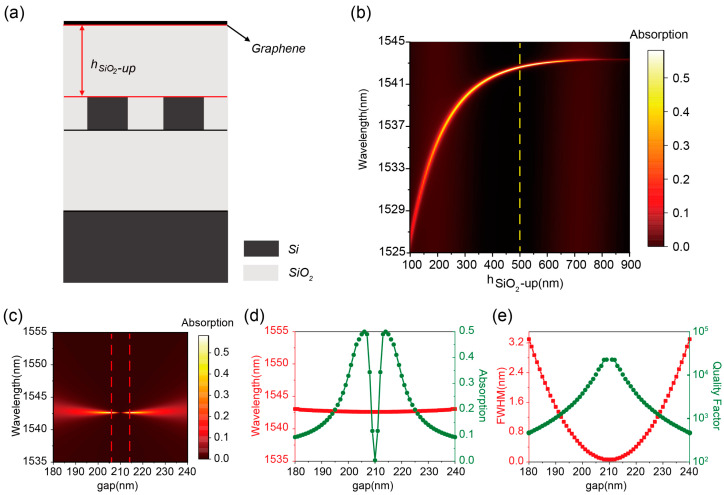
(**a**) Schematic structure of the high Q graphene-perfect absorber. (**b**) Absorption of graphene with different *h*_*SiO*_2___-*up*_ when the *gap* is kept constant at 205 nm. (**c**) Absorption of graphene with *gap* when *h*_*SiO*_2___-*up*_ is kept constant at 500 nm. (**d**) Variation of the resonance wavelength (red line) extracted from (**c**) and the absorbance (green line) with *gap*. (**e**) Variation of the resonance linewidth (red line) and the Q-factors (green line) with *gap* extracted from (**c**).

Here, a SiO_2_ dielectric layer was introduced atop the silicon dual-grating composite structure, as illustrated in Figure 2a. By modulating the thickness (*h*_*SiO*_2___-*up*_) of the upper silica layer between graphene and the composite structure, the intrinsic absorption loss (*Q_abs_*) of graphene was reduced to achieve critical coupling. The specific design process is as follows: First, a SiO_2_ dielectric layer with thicknesses spanning 100–900 nm is integrated into the composite grating structure. The results reveal that q-BIC modes persist throughout this thickness range, demonstrating spectral characteristics strongly dependent on *h*_*SiO*_2___-*up*_. The SiO_2_ layer partially preserves the out-of-plane symmetry of the composite structure, while the leakage loss exhibits a gradual reduction. Notably, the line width of the reflection spectra does not disappear because the perturbation factor (*gap* of 205 nm) that breaks the translational symmetry is still present.

According to the time-coupled mode theory (TCMT) [52], the absorption rate of the leakage mode in a two-port system can be expressed as follows:

(1) $$A=\frac{2\gamma_{0}\gamma }{{\left(w-w_{0}\right)}^{2}+{\left(\gamma_{0}+\gamma \right)}^{2}}$$

where *w* is the incident light frequency, *w*_0_ is the resonant frequency of the structure, *γ*_0_ is the dissipative loss rate, and *γ* is the radiative attenuation rate. When *w* = *w*_0_ and *γ* = *γ*_0_, the absorption of the leakage mode in the critical coupling condition can be determined to be a maximum of 50%. When the geometry of the silicon double grating is fixed in size, to achieve the critical coupling condition, it is necessary to match the absorption loss (*Q_abs_* = *w*_0_/2*γ*) with the leakage loss (*Q_rad_*) by changing the position of the graphene (i.e., the place of the graphene above the silicon double grating). When the grating gap is kept constant at 205 nm, the variation of graphene absorption with *h*_*SiO*_2___-*up*_ is shown in Figure 2b. The critical coupling condition is satisfied at *h*_*SiO*_2___-*up*_ of 540nm, achieving a graphene absorption rate of 50% as explicitly demonstrated by the yellow dashed spectral profile in Figure 2b.

While maintaining *h*_*SiO*_2___-*up*_ fixed at 500 nm, the graphene absorption spectra under varying grating gaps (180–240 nm) are calculated, as presented in Figure 2c. The vanishing resonance linewidth at 210 nm gap confirms the emergence of BIC, which indicates that the introduction of the SiO_2_ dielectric layer does not have a significant impact on the BIC. At the critical coupling conditions (206 nm and 214 nm gaps, marked by red dashed lines in Figure 2c), graphene absorption reaches 50% at 1542.61 nm wavelength. Notably, the resonance wavelength demonstrates exceptional stability against grating gap variations (Figure 2d, red curve), exhibiting significantly lower structural–parameter sensitivity compared to conventional wavelength-dependent resonant systems. This characteristic enhances its suitability for wavelength-insensitive applications, including nonlinear optics and laser engineering. As shown in Figure 2e, the 206 nm gap configuration achieves a resonance linewidth of 0.115 nm with a Q-factor reaching 1.34 × 10^4^, surpassing previous implementations in comparable systems by over an order of magnitude.

**Figure 3:**
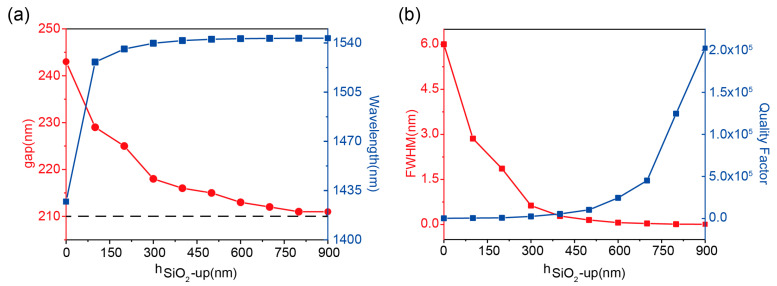
(**a**) Variation of grating spacing (red line) and resonance wavelength (blue line) with *h*_*SiO*_2___-*up*_ under critical coupling condition. (**b**) Variation of resonance linewidth (red line) and Q-factor (blue line) with *h*_*SiO*_2___-*up*_ under critical coupling condition.

These results, obtained with a 500-nm thick SiO_2_ structure, demonstrate that critical coupling conditions can be satisfied across various *h*_*SiO*_2___-*up*_ values through corresponding gap adjustments, enabling tailored graphene-perfect absorption. Specifically, independent control of leakage loss (via gap dimension) and absorption loss (via *h*_*SiO*_2___-*up*_) allows the systematic realization of graphene-perfect absorbers with tunable Q-factors. As shown in Figure 3a, the integration of SiO_2_ layers with different thicknesses above the composite grating structure, combined with optimized gap dimensions, achieves graphene-perfect absorption with distinct spectral linewidths. Notably, the required gap value for critical coupling asymptotically approaches the critical dimension *gap*_0_ as SiO_2_ thickness increases. This parameter correlation provides inherent tolerance compensation for fabrication imperfections in practical device implementations.

The resonance wavelength of the graphene-perfect absorber shown in Figure 3a shows a certain degree of redshift, consistent with Figure 2b, which is caused by a change in the effective refractive index of the composite structure. The variation of resonance linewidth and Q-factors are shown in Figure 3b, from which it can be seen that the leakage loss becomes smaller as the thickness of *h*_*SiO*_2___-*up*_ increases, resulting in the resonance linewidth gradually narrowing from 6 nm to 0.0076 nm. The Q-factor gradually becomes larger and can reach a maximum of 2.0 × 10^5^. This configuration enables a three-order-of-magnitude tunability in absorption bandwidth, ultimately achieving ultra-narrowband perfect absorption. Furthermore, the absorption bandwidth tuning range can be extended through adjustments in the graphene’s Fermi level and layer number.

**Figure 4:**
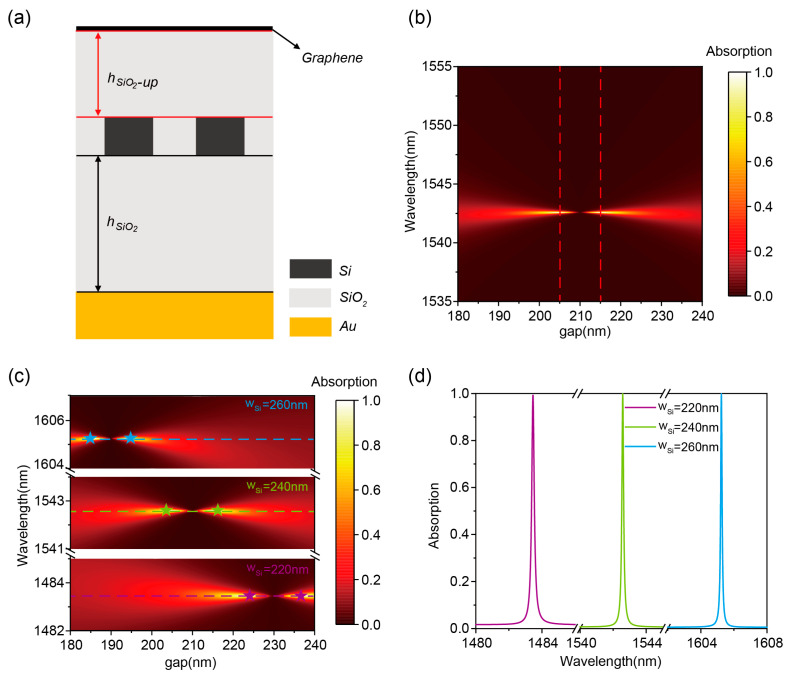
(**a**) Schematic of the structure when perfect absorption is achieved. (**b**) Absorption spectra of the graphene-perfect absorber at different *gaps*. (**c**) Absorption spectra at grating widths of 220 nm, 240 nm, and 260 nm, respectively. (**d**) Absorption spectra at different grating widths extracted from (**c**).

Notably, graphene absorption can be enhanced through the strategic integration of a back-reflector beneath the structure [53], as schematically depicted in Figure 4a. A 100 nm gold layer is introduced underneath the 1200 nm SiO_2_ as a reflector for perfect absorption, switching the two-port system to a one-port system. The absorptivity of graphene has to be rewritten as follows:

(2) $$A=\frac{4\gamma_{0}\gamma }{{\left(w-w_{0}\right)}^{2}+{\left(\gamma_{0}+\gamma \right)}^{2}}$$

Under critical coupling conditions *w* = *w*_0_ and *γ = γ*_0_, the absorption of graphene can be increased to 100%. To validate the design strategy, graphene absorption spectra under varying grating gaps (180 to 240 nm) with a fixed *h*_*SiO*_2___-*up*_ of 500 nm are calculated, as shown in Figure 4b. It is clear that the light absorption is boosted to 100% at 1542.58 nm for a gap of 205 nm. Note that the grating gaps and peak wavelengths for achieving the critical coupling condition are slightly different from the value without the bottom mirror. The underlying reason can be attributed to the varying finite thicknesses of SiO_2_ in the upper and lower portions of the nanograting, which may introduce additional factors that alter the effective refractive index of the composite structure governing the resonant wavelength shift.

Employing the same methodology, we achieved graphene-perfect absorption across 1480 nm to 1610 nm by modulating silicon grating width (*w_Si_*) while maintaining fixed structural periodicity and SiO_2_ layer thicknesses. A systematic investigation of graphene absorption dependence on grating spacing for three grating widths (220 nm, 240 nm, 260 nm) is presented in Figure 4c. The resonance wavelengths remain precisely maintained at 1483.46 nm, 1542.58 nm, and 1605.24 nm under varying width conditions, demonstrating wavelength-stabilized absorption characteristics. Corresponding absorption spectra under critical coupling conditions (pentagram markers in Figure 4c) are shown in Figure 4d, exhibiting >99.9% absorption with Q-factors of 8.0 × 10^3^, 1.2 × 10^4^, and 1.6 × 10^4^ at the respective wavelengths. Additionally, it is necessary to state that our design model can be applied to designing narrowband perfect absorbers based on multilayer graphene and other 2D materials (e.g., WSe2, MoS2). These results indicate that this system can be extended to arbitrary wavelengths across the near-infrared band.

## 4. Conclusions

In summary, we have demonstrated that perfect absorption of graphene can be achieved by using silicon nano-gratings based on the critical coupling effect of the BIC resonance. By simultaneously varying the *gap* of the nanograting and the *h*_*SiO*_2___-*up*_, the absorption bandwidth can be flexibly tuned by three orders of magnitude in the near-infrared range from 0.0076 nm to 6 nm. The *w_Si_* of 220 nm and 260 nm for graphene to achieve perfect absorption is also verified, and the strategy can be extended to an arbitrary wavelength range. The approach can be applied to a variety of critically coupled systems of 2D materials coupled to BIC meta-surfaces. The method presents a unique opportunity for designing high-Q perfect absorption with field-enhanced performance, with potential applications in nanolasers, optical switches, filters, and other advanced optoelectronic devices.

## Data Availability

The data presented in this study are available upon request.
